# Monosomy chromosome 21 compensated by 21q22.11q22.3 duplication in a case with small size and minor anomalies

**DOI:** 10.1186/s13039-018-0390-4

**Published:** 2018-08-01

**Authors:** Meng Su, Paul J. Benke, Guney Bademci, Filiz Basak Cengiz, Xiaomei Ouyang, Jinghong Peng, Carmen E. Casas, Mustafa Tekin, Yao-Shan Fan

**Affiliations:** 1 Department of Pathology and Laboratory Medicine, University of Miami Miller School of Medicine, 1601 NW 12th Avenue, Miami, FL 33136 USA; 2 Department of Genetics, Joe DiMaggio Children’s Hospital and the Charles E Schmidt College of Medicine, 1150 N 35th Avenue, Hollywood, FL 33021 USA; 3 John P. Hussman Institute for Human Genomics, University of Miami Miller School of Medicine, 1501 NW 10th Avenue, Miami, FL 33136 USA

**Keywords:** Chromosome 21, Partial monosomy, Deletion, Duplication, Partial uniparental disomy

## Abstract

**Background:**

Partial monosomy 21 is a rare finding with variable sizes and deletion breakpoints, presenting with a broad spectrum of phenotypes.

**Case presentation:**

We report a 10-month-old boy with short stature, minor anomalies and mild motor delay. The patient had a monosomy 21 and duplication of the 21q22.11q22.3 region on the remaining derivative chromosome 21 which represents a partial 21q uniparental disomy of paternal origin, upd(21q22.11q22.3)pat. The abnormalities were characterized by karyotyping, FISH, chromosomal microarray, and genotyping.

**Conclusions:**

This is the first case showing a monosomy 21 compensated by upd(21q22.11q22.3) as a mechanism of genomic rescue. Because there is no strong evidence showing imprinting on chromosome 21, the uniparental disomy itself is not associated with abnormal phenotype but has reduced phenotype severity of monosomy 21. We reviewed the previously published cases with isolated 21q deletions and identified a common deletion of 5.7 Mb associated with low birth weight, length and head circumference in the 21q21.2 region.

## Background

Full monosomy 21 has been rarely reported and is likely to be lethal in utero [1]. Even in a case of a live born with molecularly confirmed monosomy 21 [2], mosaicism could not be ruled out. It was suggested that full monosomy 21 may not exist in live born and those reported cases of monosomy 21 are likely to be either mosaicism or partial monosomy 21 resulting from a cryptic unbalanced translocation [1, 3]. Some cases previously reported were identified prior to the era of high resolution G-banding and molecular cytogenetics. Several cases described as full monosomy 21 initially by conventional karyotyping were later re-classified as partial monosomy by molecular cytogenetics or other molecular techniques [4–6]. Among the more than 30 cases previously reported with a partial monosomy 21, about half were isolated chromosome 21 segmental monosomy without other abnormalities identified [7, 8]. The remaining half had rearrangements involving other chromosomes in addition to chromosome 21 [7, 9–14]. Each partial monosomy 21 case represents a rare and unique finding with variable deletion breakpoints, and therefore the cases with partial monosomy 21 have a broad spectrum of phenotypes.

Here, we present a patient with monosomy 21 and a duplication in the 21q22.11q22.3 region on the remaining derivative chromosome 21 which represent a partial uniparental disomy (UPD), with discussions on the possible mechanisms with which the abnormality arose in this case and the genotype-phenotype correlation of 21q deletions.

## Methods

### DNA extraction and chromosomal microarray analysis (CMA)

CMA was performed using a combined comparative genomic hybridization (CGH) and single nucleotide polymorphism (SNP) microarray platform SurePrint G3 4x180K CGH + SNP microarray chip (Agilent). The CMA platform is composed of approximately 120,000 oligonucleotide probes for CGH analysis and 60,000 oligonucleotide probes for SNP analysis with an average resolution of 5~ 10 Mb for absence of heterozygosity (AOH)/UPD detection. Probes were annotated against NCBI Build 37 (UCSC hg19, February 2009). The data was analyzed using Agilent C microarray scanner and CytoGenomics Edition 4.0.2.21 software.

### Chromosome analysis

Peripheral blood samples of the patient and his parents were cultured and slides were prepared according to standard laboratory protocol. Chromosome analysis was performed on 20 G-banded metaphase cells at the resolution level of 500–550 bands per haploid set, using standard technology. Chromosome abnormalities were described using An International System for Human Cytogenomic Nomenclature [15].

### Fluorescence in-situ hybridization (FISH)

FISH was performed on metaphase chromosome slides according to a protocol recommended by the manufacturer of the FISH probes used in this study. A mixture of AneuVysion Vysis locus-specific indicator (LSI) 13 and 21 probes (Abbott, Abbott Park, IL) was used and these two probes were designed to hybridize to the 13q14 and the 21q22.13q22.2 regions respectively. 10 metaphases and 200 interphase cells were analyzed.

### Genotyping

Genotyping was done by Sanger sequencing on DNA samples of the patient and his parents. Briefly, genomic DNA was extracted and purified from peripheral blood samples using QIAamp DNA Blood Midi Kit (Qiagen). Primers specific for four highly polymorphic single nucleotide polymorphisms (SNPs) (rs2776109, rs68172960, rs6517210, and rs9968008) across the chromosome 21 were designed using Primer3 (http://primer3.ut.ee/, version 4.0.0) and UCSC In-Silico PCR (University of California Santa Cruz, Santa Cruz, CA). The targeted regions were amplified with a touchdown thermal cycling program, which was 3 min at 94 °C followed by 5 s at 94 °C; 30 s at 65 °C (minus 2 °C every two cycles); 60 s at 72 °C, then 7 min at 72 °C. The PCR products were purified using SOPE resin (Edge Biosystems, Gaithersburg, MD) combined with Sephadex Plate. Afterward, the purified amplicons were bidirectionally sequenced using BIG DYE Terminator Ready Reaction Mix v3.1 on an ABI 3130 system (Applied Biosystems, Foster City, CA). The results were analyzed using Sequencher (version 5.4.5, Ann Arbor, MI).

## Case presentation

An Hispanic male infant was referred to genetics clinic at three months of age by his neurologist for short stature and minor facial findings. A prenatal ultrasound scan at 20 weeks of gestation showed intrauterine growth restriction (IUGR). Labor was medically induced at 38 weeks because of the small size and an emergency Caesarean section was performed due to heart deceleration during induction. His weight was 2.183 kg (< 1%ile), length was 40.5 cm (< 1%ile), and head circumference was 32.5 cm (1%ile) at birth. He was otherwise healthy and discharged from the hospital after 3 days. By twelve months of age, his length was 68.6 cm (< 1%ile) and weight was 7.7 kg (2%ile), but head circumference had increased dramatically and reached 45.7 cm (36%ile).

**Fig. 1:**
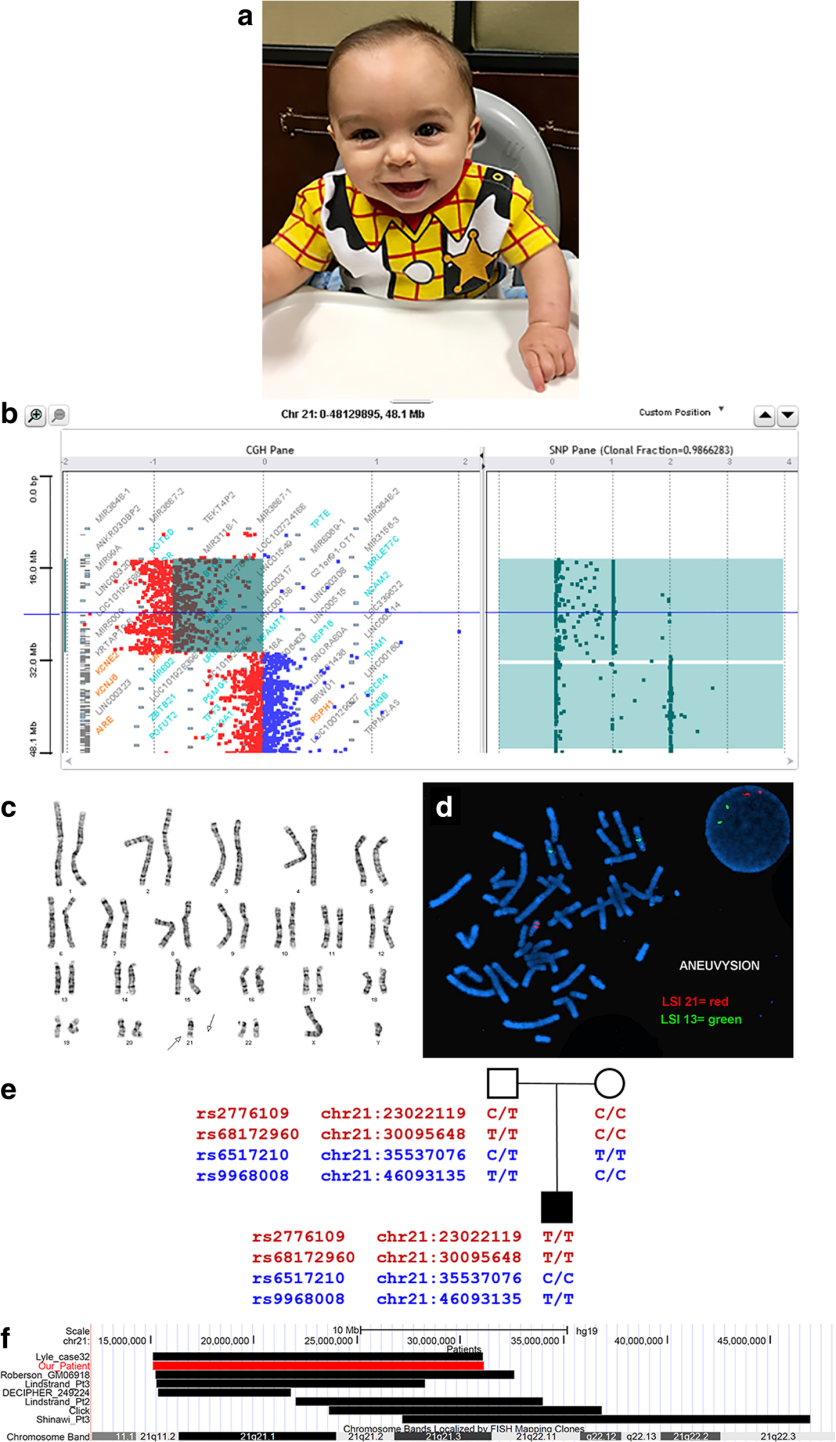
Clinical presentation and molecular cytogenetics analysis of the present patient. **a** Clinical presentation of the patient at 8 months of age. He had minor facial dysmorphic features including a broad, prominent forehead, mildly depressed nasal bridge, thin upper lip, small chin, and boarder-line low set ears. **b-d** Cytogenetic analysis identified a deletion and an AOH resulted by duplication in chromosome 21. **b** CGH and SNP array profile of chromosome 21. The numbers in the CGH pane are indicating log2 ratio of the intensity of fluorescence of the patient versus control genome. In the SNP pane, the numbers indicate the number of B allele. **c** Karyotype of the patient. **d** FISH analysis in both metaphase and interphase cells using probes designed for testing presence or absence of the 21q22.11q22.3 region (red). **e** Genotyping by Sanger sequencing indicating the paternal origin of the derivative chromosome 21. SNPs rs2776109 and rs68172960 (shown in red) are located in the 21q11.2q21.3 region, and rs6517210 and rs9968008 (shown in blue) covered the 21q22.11q22.3 UPD region. **f** Genomic location of the 21q deletions in the present patient and previously published cases. The red bar indicates the 15.98 Mb deletion at 21q11.2q21.3 (chr21:15,143,552_31,118,908, GRCh37/hg19) in our patient. The black bars represent the deletion regions of previously reported cases with isolated deletion at 21q overlapping with our patient, without any other chromosomal structural abnormality. Genomic locations are showed by UCSC genome browser

The patient’s bone age was delayed. At 11 months of age, his bone age was between three and six months. His motor skills were mildly delayed about two to three months. At four months of age, he was mildly hypertonic and was late lifting up. His left eye had difficulty with upward gaze, and he had problems tracking objects. At eight months of age, he was able to sit with support and grab at objects. He could roll front to back, but not yet back to front. He had shoulder stiffness and difficulty reaching his arms over his head. He had minor facial dysmorphic features including a broad, prominent forehead, mildly depressed nasal bridge, thin upper lip, small chin, and boarder-line low set ears (Fig. 1a). He was happy, smiling, engaged, and had good eye contact. His mental development was normal for age. Both his 35 years old mother and 47 years old father were healthy and did not show similar findings.

The CGH array detected a 15.98 Mb deletion in the 21q11.2q21.3 chromosome region, arr[GRCh37] 21q11.2q21.3(15143552_31118908)× 1 (Fig. 1b). The SNP array confirmed the deletion and also showed absence of heterozygosity (AOH) at 21q22.11q22.3 with a size of approximately 14.32 Mb (chr21:33,080,313_47,399,375, GRCh37/hg19) (Fig. 1b). Chromosome analysis revealed an abnormal karyotype, 45,XY,-21,der(21)dup(21)(q22.11q22.3) (Fig. 1c), that is a loss of an entire chromosome 21 and duplication of the 21q22.11q22.3 region on the derivative 21. This duplication was subsequently confirmed by FISH showing the two signals on the der(21) (Fig. 1d). Chromosome analysis by G-banding demonstrated a normal karyotype in both parents. Genotyping clearly demonstrated paternal origin of the der(21) (Fig. 1e).

## Discussion and conclusions

Combining the results of chromosome analysis, FISH, CGH/SNP microarray and genotyping, this patient has a single copy of the 21q11.2q21.3 region with a size of approximately 16 Mb, and two copies of the remaining chromosome 21q22.11q22.3 region, resulting from loss of one entire chromosome 21 and duplication of the 21q22.11q22.3 in the derivative 21 chromosome. The derived 21 is paternal in origin as shown by genotyping, representing a partial UPD in 21q22.11q22.3 region, upd(21q22.11q22.3)pat. This partial UPD in the der(21) has compensated for the loss of an entire chromosome 21, thus leading to a mildly abnormal phenotype.

Among the over 30 patients of partial monosomy 21 published previously, none of them share common breakpoints of their deletion regions, and their phenotype shows a broad spectrum [7, 12, 14]. Therefore, each partial monosomy 21 case is a unique finding and more patients are warranted to further elucidate the genotype-phenotype correlation and unveil the possible consequences of the deletions. To date, this is the first patient reported with a monosomy 21 associated with a partial upd(21)pat in the most gene-rich region [16]. Because there is no strong evidence showing imprinting on chromosome 21 and upd(21) is not associated with an abnormal phenotype [17, 18], the partial upd(21), upd(21q22.11q22.3)pat in our case has reduced phenotype severity of monosomy 21.

A well accepted model proposed by Lyle et al. [12] divided chromosome 21 into three regions: Region 1 (~ 31.2 Mb, 21q11.2-q22.11) harboring more than 50 genes, Region 2 (31.2–36 Mb, 21q22.11-q22.12) containing more than 80 genes, and Region 3 (~ 36–37.5 Mb to 21qter, 21q22.12-q22.3) encompassing more than 130 genes. Phenotypic severity associated with these three regions are severe, lethal, and mild, respectively. In the present patient, the missing of an entire chromosome 21 and the duplication on the derivative chromosome 21 have resulted in a deletion of about 16 Mb in the 21q11.2q21.3 region, which is within Region 1. Lyle et al. [12] proposed that deletions of Region 2 lead to a much more severe phenotype and are not compatible with survival. Nonetheless, Roberson et al. [14] suggested to expand Region 1 to merge with Region 2, because four patients they reported along with one patient reported by Lindstrand et al. [19] and three DECIPHER cases (DECIPHER patient 2609, 4976, 249,393) all had deletions overlapping part of Region 2. The severity of clinical presentation for deletions in this region was variable and seems comparable with that of Region 1. However, even though the above-mentioned cases had deletions overlapping part of Region 2, there were no non-mosaic cases reported that spanned the entire Region 2. There was one case in the literature with a deletion spanning Region 2 which was 15% mosaic [20]. Deletion of Region 3 is associated with a mild phenotype. In the present patient, there is no deletion in Region 2 and Region 3. Instead, there is a UPD in the derivative chromosome 21 that spans the whole Region 2 and Region 3.

We suggest that monosomy rescue is the most likely mechanism of the chromosome abnormalities observed in our patient. One possibility is that a normal sperm fertilized an ovum with nullisomy 21, and then the paternal chromosome 21 duplicated its q22.1q23.3 region in the zygote. The q22.1q23.3 region contains the most biologically important genes to compensate the loss of an entire maternal chromosome 21. The second possibility is a somatic event: A loss of the maternal chromosome 21 occurred in the first mitotic cell division after a normal fertilization, followed by duplication of the q22.1q23.3 region in the paternal chromosome 21. The phenomenon of monosomy rescue supports the hypothesis that a deletion spanning the gene dense region in chromosome 21 cannot be tolerated [12] and a pure monosomy 21 cannot exist in live born [1, 3]. Our findings re-emphasize the importance of Region 2 in chromosome 21.

Genotype-phenotype correlation in patients with partial monosomy 21 has always been challenging because of the variable sizes of the deletions [7]. Such correlation can be more complicated due to presence of complex abnormalities such as unbalanced rearrangements involving other chromosomes which are usually associated with more severe phenotypes and are more complex to interpret [12, 14, 19].

Among previously published cases with isolated chromosome 21 segmental monosomy without other abnormalities, we compared their breakpoints with our patient and discovered seven of them had deletions overlapped with the deletion region observed in our patient (Fig. 1f). The phenotypes of these seven patients and our patient are summarized in Table 1. Our patient shares some of the most common abnormal phenotypes observed in this group (Table 1). The patient reported by Lyle et al. (case 32) [12] had a deletion similar to that of the present case. The common features of these two patients include developmental delay and microcephaly. The 21q21.2 region with a size of 5.7 Mb (chr21: 22,560,763 to 28,265,000) encompassing 22 genes was commonly deleted in 4 of these 7 patients (Patient 2 and 3 in Lindstrand’s report, the case reported by Click et al., and the present case). All these 4 patients had low birth weight, length and head circumference. It was noted that the weight and length of the Patient 3 in the Lindstrand’s report reached to the normal range at 5 years and 10 months of age. In our patient at 12 months of age, no obvious improvement was observed in the measurements besides that of head circumference. These observations may suggest association of 21q21.2 deletion with a low birth weight, length and head circumference.

**Table 1:** Summary of phenotypes of isolated chromosome 21 deletion patients with overlapping regions with our patient

	Present patient	Lyle case 32	Lindstrand Pt 2	Lindstrand Pt 3	Roberson GM06918	Click	Shinawi Pt 3	DECIPHER 249224	# of abnormal phenotypes
intellectual disability		yes	yes	yes	yes		yes		5
low birth weight	yes		yes	yes		yes			4
broad or depressed nasal bridge		yes			yes	yes	yes		4
short stature	yes	yes			yes				3
low anterior or posterior hairline		yes			yes	yes			3
low set ears	yes		yes			yes			3
downward slanting palpebral fissures			yes		yes	yes			3
hypertelorism	yes		yes				yes		3
congenital heart defect			yes	yes		yes			3
delayed speech and language development				yes				yes	2
hypotonia	yes	yes							2
microcephaly	yes	yes							2
large ears		yes				yes			2
high or cleft palate		yes			yes				2
strabismus						yes	yes		2
feeding difficulties			yes						1
hypertonia		yes							1
distal limbs abnormalities				yes					1

Abnormal phenotypes are listed from the most common to least common ones

Intellectual disability and congenital heart malformations are the most common features in partial monosomy 21 but were not found in our patient. Previous studies have suggested association of intellectual disability with the *ITSN1* gene, and proposed *KCNE1, RCAN1, CLIC6* and *RUNX1* as candidate genes for congenital heart malformations [19]. All of these genes are located in Region 2 and none was lost in our case.

In summary, the deletion in the present case encompasses 63 genes, and none of them are known dosage sensitive genes. The 15.98 Mb deletion in the 21q11.2q21.3 region is caused by loss of the entire maternal chromosome 21 which was compensated by a 14.32 Mb paternal duplication of the 21q22.11q22.3 region in the derivative chromosome 21. Although the deletion is within the 21q Region 1 (q11.2q22.1), the smaller deletion is associated with relatively mild developmental disabilities. Deletion of the 21q21.2 region is associated with a low birth weight and short stature as shown by several reported cases.

## Abbreviations

AOH: Absence of heterozygosity
CGH: Comparative genomic hybridization
CMA: Chromosomal microarray analysis
FISH: Fluorescence in-situ hybridization
IUGR: Intrauterine growth restriction
SNP: Single nucleotide polymorphism
UPD: Uniparental disomy
